# Enhanced Patient Education for Colonic Polyp and Adenoma Detection: Meta-Analysis of Randomized Controlled Trials

**DOI:** 10.2196/17372

**Published:** 2020-06-01

**Authors:** Xu Tian, Ling-Li Xu, Xiao-Ling Liu, Wei-Qing Chen

**Affiliations:** 1 Chongqing University Cancer Hospital Chongqing China

**Keywords:** colonoscopy, bowel preparation, patient education, polyp detection rate, adenoma detection rate, meta-analysis

## Abstract

**Background:**

To improve patients’ comprehension of bowel preparation instructions before colonoscopy, enhanced patient education (EPE) such as cartoon pictures or other visual aids, phone calls, mobile apps, multimedia education and social media apps have been proposed. However, it is uncertain whether EPE can increase the detection rate of colonic polyps and adenomas.

**Objective:**

This meta-analysis aimed to evaluate the efficacy of EPE in detecting colonic polyps and adenomas.

**Methods:**

We searched PubMed, EMBASE, and Cochrane Central Register of Controlled Trials from their inception to June 2019 for the identification of trials comparing the EPE with standard patient education for outpatients undergoing colonoscopy. We used a random effects model to calculate summary estimates of the polyp detection rate (defined as the number of patients with at least one polyp divided by the total number of patients undergoing selective colonoscopy), adenoma detection rate (defined as the number of patients with at least one adenoma divided by the total number of patients undergoing selective colonoscopy), advanced adenoma detection rate (defined as the number of patients with at least one advanced adenoma divided by the total number of patients undergoing selective colonoscopy), sessile serrated adenoma detection rate (defined as the number of patients with at least one sessile serrated adenoma divided by the total number of patients undergoing selective colonoscopy), cancer detection rate (defined as the number of patients with at least one cancer divided by the total number of patients undergoing selective colonoscopy), or adenoma detection rate - plus (defined as the number of additional adenomas found after the first adenoma per colonoscopy). Moreover, we conducted trial sequential analysis (TSA) to determine the robustness of summary estimates of all primary outcomes.

**Results:**

We included 10 randomized controlled trials enrolling 4560 participants for analysis. The meta-analysis suggested that EPE was associated with an increased polyp detection rate (9 trials; 3781 participants; risk ratio [RR] 1.19, 95% CI 1.05-1.35; *P*<.05; I^2^=42%) and adenoma detection rate (5 trials; 2133 participants; RR 1.37, 95% CI 1.15-1.64; *P*<.001; I2=0%), which were established by TSA. Pooled result from the inverse-variance model illustrated an increase in the sessile serrated adenoma detection rate (3 trials; 1248 participants; odds ratio 1.76, 95% CI 1.22-2.53; *P*<.05; I^2^=0%). One trial suggested an increase in the adenoma detection rate - plus (RR 4.39, 95% CI 2.91-6.61; *P*<.001). Pooled estimates from 3 (1649 participants) and 2 trials (1375 participants) generated no evidence of statistical difference for the advanced adenoma detection rate and cancer detection rate, respectively.

**Conclusions:**

The current evidence indicates that EPE should be recommended to instruct bowel preparation in patients undergoing colonoscopy because it can increase the polyp detection rate, adenoma detection rate, and sessile serrated adenoma detection rate. However, further trials are warranted to determine the efficacy of EPE for advanced adenoma detection rate, adenoma detection rate - plus, and cancer detection rate because of limited data.

## Introduction

### Background

Colorectal cancer (CRC) is the third most common cancer and the second cause of cancer-related mortality among both sexes worldwide, with 1.8 million new cases and 0.88 million deaths in 2018 [1]. Colonoscopy is recommended as the principal approach for decreasing CRC incidence and associated mortality by detecting and then removing the precancerous lesions [2-5]. However, adequate bowel preparation is the prerequisite for a successful colonoscopy [6]. Evidence revealed that inadequate bowel preparation was associated with increased risk of missing colonic lesions, prolonged procedural time, and lower cecal intubation rate [7,8]. Issued data suggested approximately 18% to 30.5% of inadequate bowel preparation in patients undergoing colonoscopy [9-11]. Therefore, it is particularly urgent to apply an effective intervention to improve the quality of bowel preparation [12].

Previous studies have determined various factors that were linked to the quality of bowel preparation, such as the type of diet restriction, type of colon cleansing solutions ingested, methods of ingesting the solution, and patient’s adherence to the solution [10,13-15]. Adequate comprehension of details of instructions is a major contributor to the quality of colon cleansing because bowel preparation is very complex [16]. Patients usually receive written booklet and/or verbal instructions from professionals before colonoscopy for bowel preparation and dietary restriction, which are defined as standard patient education [2]. However, the effect of standard patient education on bowel preparation is not enough [10]. To improve the patient’s comprehension of bowel preparation instructions, enhanced patient education (EPE; such as cartoon pictures, phone calls, mobile apps, and social media apps) has been proposed and then tested [2]. So far, several meta-analyses have evaluated the efficacy of EPE in improving the quality of bowel preparation and demonstrated an improvement [2,16-19]. However, evidence revealed that adequate bowel preparation provides good colonoscopy vision and thus increases the detection rate of colonic polyps and adenomas [20-23]. The fact that 2 meta-analyses evaluated the efficacy of EPE interventions to detect polyps and both did not find significant differences is discouraging [2,17]. As a result, the magnitude of benefit of EPE interventions in detecting colonic polyps and adenomas remains uncertain. It is noteworthy that recently, several randomized controlled trials (RCTs) reporting conflicting results have been published. More importantly, as most CRCs transform from polyps and adenomas, early detection and then removal of premalignant colonic polyps and adenomas is crucial [19]. Thus, as one of the most important colonoscopy quality metrics, the detection of colonic polyps and adenomas should be primarily measured and evaluated [24].

### Objective

The aim of this meta-analysis was to evaluate the efficacy of EPE interventions in detecting CRC precursor polyps and adenomas compared with standard patient education.

## Methods

### Methodological Standard

We conducted this meta-analysis according to the methods proposed by the Cochrane Collaboration [25] and reported the pooled estimates in accordance with the framework proposed by the Preferred Reporting Items for Systematic Reviews and Meta-Analysis statement [26]. There was no formal protocol for this meta-analysis.

### Search Strategy

A systematic search was performed in PubMed, EMBASE, and the Cochrane Central Register of Controlled Trials from their inception to June 2019 for the identification of relevant RCTs. All search strategies were built using Exploded Medical Subject Headings and the appropriate corresponding text words. Language and status of publication were not restricted. We have summarized the details of the full search strategy in Multimedia Appendix 1. We updated the search results on August 10, 2019. The bibliographies of previous meta-analyses and eligible studies were also manually checked to identify additional potentially eligible studies.

### Study Selection

We used the following inclusion criteria to enroll any eligible studies in this study: (1) all adult participants aged more than 18 years who were instructed to receive elective outpatient colonoscopy, regardless of morning and afternoon colonoscopy; (2) the patients assigned in the study group were instructed with EPE regimes, and the ones enrolled in the control group were instructed with standard patient education regimes; (3) the eligible study design was RCTs; however, an abstract with sufficient information was also considered; and (4) studies published in English. Two investigators (XT and LX) independently searched citations; excluded duplicates; checked the titles and abstracts for eligibility; and then categorized the studies as included, excluded, or requiring further full-text assessment. We excluded duplicates with poor quality or relatively insufficient data. We also excluded conference abstracts without sufficient information. A third senior investigator (WC) was consulted for a final decision if there was any disagreement between the 2 investigators.

### Data Extraction

Two independent investigators (XT and LX) were assigned to use a standardized Word (version 2013, Microsoft Office, Microsoft Corporation) table to extract essential data, and then, they completed the cross-checking of corresponding results. The following data were extracted: basic characteristics of eligible trials including leading author, publication year, country, and financial sources; risk of bias criteria based on the Cochrane Collaboration risk of bias tool [27]; and clinical characteristics including age, sex, sample size, indication for colonoscopy, details of diet restriction and colon cleansing solutions, details of education interventions, and outcomes of interest. A third senior investigator (WC) was consulted for a final decision if there was any disagreement between the 2 investigators.

### Outcome Variables and Definitions

We defined the colonic polyp detection rate (PDR) and adenoma detection rate (ADR) as the primary outcomes, which were defined as the number of patients with at least one polyp or adenoma divided by the total number of patients undergoing selective colonoscopy. We considered the advanced adenoma (defined as adenoma ≥10 mm) detection rate (AADR), sessile serrated adenoma detection rate (SSADR), and cancer detection rate (CDR) as secondary outcomes, which were defined as the number of patients with at least one advanced adenoma, sessile serrated adenoma, or cancer divided, respectively, by the total number of patients undergoing selective colonoscopy. We also considered ADR-plus, which was defined as the number of additional adenomas found after the first adenoma per colonoscopy [28], and the right and left colon polyp and ADR, which was defined as the number of patients with at least one right and left colon polyp and adenoma divided by the total number of patients undergoing selective colonoscopy, as secondary outcomes.

### Assessment of Risk of Bias

We assigned 2 independent investigators (XT and XL) to appraise the risk of bias with the Cochrane risk of bias tool (the Cochrane Collaboration) [27]. An individual trial would be labeled as *low*, *unclear*, or *high* risk of bias according to the following criteria: random sequence generation, allocation concealment, blinding of participants and personnel, blinding of outcome assessment, incomplete outcome data, selective reporting, and other bias. A third senior investigator (WC) was consulted for a final decision if there was any disagreement between the 2 investigators. Following the recommendations proposed by the Cochrane Collaboration, a trial was considered a *high-level* trial when all key domains are rated as having a *low* risk of bias, a trial was considered a *low-level* trial when any one or more key domains are rated as having a *high* risk of bias, and otherwise, a trial was considered a *moderate-level* trial.

### Data Analysis

We expressed summary estimates as a risk ratio (RR) or odds ratio (OR) with 95% CI. Heterogeneity was measured by the I^2^ statistic, which describes the percentage of total variation across studies that is due to heterogeneity rather than chance [29]. We performed all statistical analyses using random effects model regardless of heterogeneity. In addition, subgroup analyses for the primary outcomes were conducted according to geographical regions (Western vs Asian) and indications (screening vs diagnostic vs mixed). We also conducted subgroup analysis for primary outcomes according to the administration method of ingesting colon cleans solutions (single dose vs split dose) because Zawaly et al [30] demonstrated that split dose compared with single dose was associated with increased detection of adenomas and advanced adenomas. *P*<.05 was considered statistically significant, except where otherwise specified. All statistical analyses were performed using Cochrane Review Manager (RevMan, version 5.3.5, 2014; the Nordic Cochrane Centre, the Cochrane Collaboration) [31]. For pooled estimates of SSADR, we used inverse-variance statistic due to various data reported in analyzed trials, and for remaining pooled estimates, we used the Mantel-Haenszel model. An RR or OR value greater than 1 indicates that there was a higher detection rate of the specified colonic polyp and adenoma. We planned to assess publication bias if any pooled group consisted of 10 or more trials [32].

### Trial Sequential Analysis

The magnitude of efficacy of summary estimates from cumulative meta-analyses and the risk of type I error are susceptible to repetitive hypothesis test of accumulating scarce information [33]. Thus, trial sequential analysis (TSA), which has the potential of constantly adjusting the significance level and then drawing monitoring boundaries and calculating adjusted information size, was proposed to address issues faced by the traditional meta-analysis [34-36]. The conclusion is conclusive if the accumulative sample size is more than the adjusted information size and the Z-curve is across the trial sequential monitoring boundary or futility boundary. We conducted TSA to test the robustness of summary estimates of primary outcomes according to an alpha error of .05, a beta error of .20 (a power of 80%), and an anticipated intervention effect of 20% relative risk reduction using TSA version 0.9 beta (Copenhagen Trial Unit, Center for Clinical Intervention Research) [37,38].

## Results

### Literature Search

**Figure 1:**
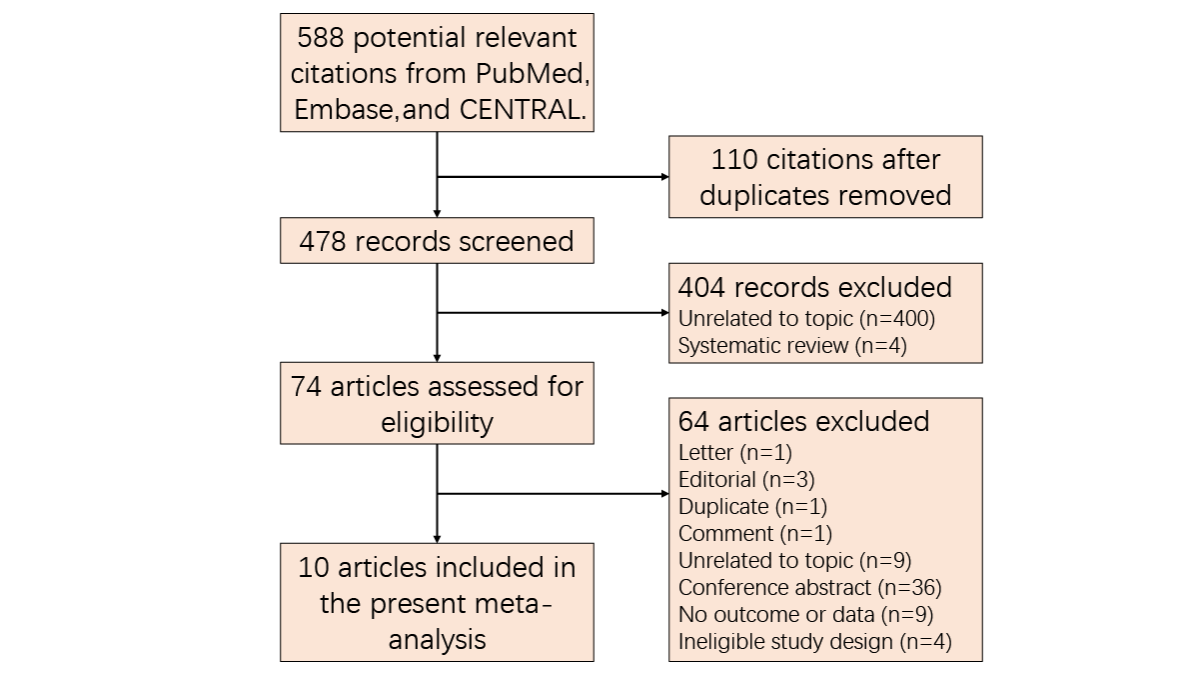
Retrieval and selection of RCTs for the meta-analysis. CENTRAL: Cochrane Controlled Register of Trials; RCT: randomized controlled trial.

Figure 1 depicts the retrieval and selection of records. Initial search captured 588 records in 3 targeted databases. All records were imported to EndNote software (Thomson Reuters), and then, we deleted 110 duplicate records after running the finding duplicates function. We excluded additional 404 records after checking the titles and abstracts because of the following reasons: systematic review and meta-analysis and irrelevant to the analysis. We omitted 64 studies after carefully double-checking the full text in the remaining 74 studies because of the following reasons: 9 articles investigated a topic unrelated to this study, 1 article was a letter to the editor, 3 articles were editorials, 1 article was a duplicate publication, 1 article was a comment on published article, 36 were conference abstracts without sufficient information, 9 articles did not report essential outcomes or data that were considered in our study, and 4 articles used ineligible study design. We, thus, included 10 eligible RCTs in the final meta-analysis after checking the full text for eligibility [9,39-47].

### Study Characteristics

We document the details of basic characteristics of the 10 eligible RCTs in Table 1. All trials were reported between 2011 and 2018. The sample size in individual trial ranged from 94 to 969 (a total of 4560 participants). Of the 10 eligible RCTs, 3 [39,41,46] were from Western countries, including the United States [39,41] and Germany [46], and 7 [9,40,42-45,47] were from Asian countries, including China [9,43,47], Korea [40,45], and South Korea [42,44]. In total, 8 RCTs [9,39-41,43-46] were designed with two arms and remaining 2 RCTs [42,47] with three arms. The participants in 5 RCTs [39,40,42,44,45] received screening colonoscopy; in 4 RCTs [9,41,43,46] received screening, surveillance, or diagnostic colonoscopy; and in 1 RCT [47] received diagnostic colonoscopy. A total of 8 RCTs [9,40-44,46,47] described the details of diet restriction, and other 2 RCTs [39,45] did not report on the diet. Of the 10 included RCTs, 9 reported PDR as outcome [39-47], 5 reported ADR as outcome [9,41,42,46,47], 3 reported AADR as outcome [9,46,47], 3 reported SSADR as outcome [9,41,46], 2 reported CDR as outcome [9,43], and 1 reported ADR-plus as outcome [46].

### Risk of Bias

Details of the risk of bias of individual trial are summarized in Table 2: 7 trials were rated as low level [39-42,44-46], 2 were moderate level [9,43], and 1 was high level [47]. A total of 8 trials appropriately generated randomization sequence [9,39,41-43,45-47], and 5 trials correctly conducted allocation concealment [9,42,43,46,47]. All trials [9,40-47] appropriately blinded endoscopist and reported anticipated outcomes.

**Table 1:** Details of studies included in this meta-analysis.

Study	Country	Sample size (SPE^a^/EPE^b^)	Sex (male/female; SPE/EPE)	Education strategies		Indications	Bowel cleansing regimen	Diet restriction	Start time of education	Outcomes
				SPE	EPE					
Calderwood et al (2011) [39]	United States	969 (492/477)	(205/287; 198/279)	Standard written precolonoscopy information	Visual aid	Screening colonoscopy	4 L PEG^c^ alone or plus bisacodyl	NR^d^	NR	PDR^e^
Cho et al (2017) [40]	Korea	142 (71/71)	(42/29; 42/29)	Verbal and written instructions	Smartphone app	Screening colonoscopy	2 L PEG plus ascorbate with single dose	Low residue	3 days before	PDR
Garg et al (2016) [41]	United States	94 (46/48)	(21/21; 21/27)	Standard written precolonoscopy information	Multimedia education	Screening or surveillance colonoscopy	NR (single dose)	Clear liquid	NR	PDR, ADR^f^, and SSADR^g^
Kang et al (2016) [9]	China	770 (383/387)	(191/192; 202/185)	Verbal and written instructions	WeChat	Mixed^h^	4 L PEG 4000 with split dose	Clear liquid	15 days before	ADR, AADR^i^, SSADR, and CDR^j^
Lee et al (2015) [42]	South Korea	394 (137/255)	(73/64; 155/98)	Verbal and written instructions	Telephone or SMS reminder	Screening colonoscopy	2 L PEG plus ascorbic acid with split dose	Low residue	2 days before	PDR and ADR
Liu et al (2013) [43]	China	605 (300/305)	(147/153; 160/145)	Verbal and written instructions	Telephone re-education	Mixed	2 L PEG 4000 or 1.5 L sodium phosphate with single dose	Clear liquid	1 day before	PDR and CDR
Park et al (2016) [44]	South Korea	502 (252/250)	(167/85; 157/93)	Regular instruction	Educational video	Screening colonoscopy	2 L PEG with split dose	Clear liquid	1 day before	PDR
Tae et al (2012) [45]	Korea	205 (103/102)	(71/32; 73/29)	Verbal and written instructions	Cartoon visual aids	Screening colonoscopy	PEG with split dose	NR	NR	PDR
Walter et al (2018) [46]	Germany	495 (247/248)	(116/131; 126/122)	Standard education	SMS	Mixed	2 L PEG plus ascorbic acid with split dose	Low fiber	4 days before	PDR, ADR, AADR, and SSADR
Wang et al (2018) [47]	China	384 (127/257)	(68/59; 149/108)	Verbal and written instructions	WeChat or SMS	Diagnostic colonoscopy	3 L PEG with split dose	Clear liquid	2 days before	PDR, ADR, ADR-plus, and AADR

^a^SPE: standard patient education.

^b^EPE: enhanced patient education.

^c^PEG: polyethylene glycol.

^d^NR: not reported.

^e^PDR: polyp detection rate.

^f^ADR: adenoma detection rate.

^g^SSADR: sessile serrated adenoma detection rate.

^h^Mixed represents the combination of diagnostic, screening, and surveillance colonoscopy.

^i^AADR: advanced ADR.

^j^CDR: cancer detection rate.

**Table 2:** Details of quality assessment of eligible studies using the Cochrane risk of bias tool.

Study	Random sequence generation	Allocation concealment	Blinding of participants and personnel	Blinding of outcome assessment	Incomplete outcome date	Selective reporting	Other bias	Overall level
Calderwood et al (2011) [39]	Low risk	Unclear risk	Low risk	High risk	Low risk	Low risk	Low risk	Low level
Cho et al (2017) [40]	High risk	High risk	Low risk	High risk	Low risk	Low risk	Low risk	Low level
Garg et al (2016) [41]	Low risk	Unclear risk	Low risk	High risk	High risk	Low risk	Low risk	Low level
Kang et al (2016) [9]	Low risk	Low risk	Low risk	Unclear risk	Low risk	Low risk	Low risk	Moderate level
Lee et al (2015) [42]	Low risk	Low risk	Low risk	High risk	Low risk	Low risk	Low risk	Low level
Liu et al (2013) [43]	Low risk	Low risk	Low risk	Unclear risk	Low risk	Low risk	Low risk	Moderate level
Park et al (2016) [44]	Unclear risk	Unclear risk	Low risk	High risk	Low risk	Low risk	Low risk	Low level
Tae et al (2012) [45]	Low risk	Unclear risk	Low risk	High risk	High risk	Low risk	Low risk	Low level
Walter et al (2018) [46]	Low risk	Low risk	Low risk	High risk	Low risk	Low risk	Low risk	Low level
Wang et al (2018) [47]	Low risk	Low risk	Low risk	Low risk	Low risk	Low risk	Low risk	High level

### Primary Outcomes

**Figure 2:**
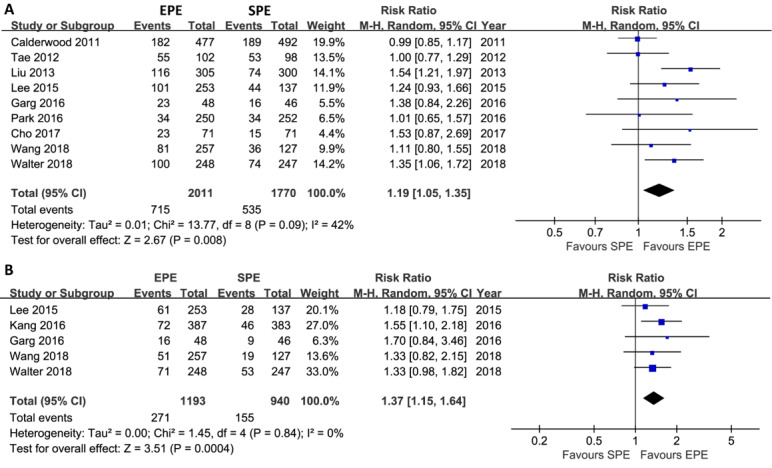
Meta-analysis of the effect of EPE on PDR (A) and ADR (B). This pooled result indicated a statistical difference regarding PDR and ADR between EPE and SPE groups. The summary effect estimates (risk ratio, RR) for individual randomized controlled trial (RCT) are indicated by blue rectangles (the size of the rectangle is proportional to the study weight), with the black horizontal lines representing 95% CIs. The overall summary effect estimate (RR) and 95% CI are indicated by the black diamond below. EPE: enhanced patient education; SPE: standard patient education; PDR: polyp detection rate; ADR: adenomas detection rate; RR: risk ratio; and M-H: Mantel-Haenszel.

Figure 2 depicts the summary results of primary outcomes. Meta-analysis based on a random effects model suggested an increase in the detection rate of polyps (9 trials; 35.55% [715/2011] of participants vs 30.23% [535/1770] of participants; RR 1.19, 95% CI 1.05-1.35; *P*=.008; I^2^=42%) and adenomas (5 trials; 22.72% [271/1193] of participants vs 16.5% [155/940] of participants; RR 1.37, 95% CI 1.15-1.64; *P*<.001; I^2^=0%) in patients undergoing EPE. Subgroup analyses indicated that EPE increased the PDR in Asian patients; in patients ingested solutions with split dose and single dose; and in mixed patients undergoing screening, surveillance, and diagnostic colonoscopy, and that EPE increased the ADR in all patients regardless of geographical regions and in patients ingested solutions with split dose. The summary of subgroup analyses is shown in Multimedia Appendix 2.

**Figure 3:**
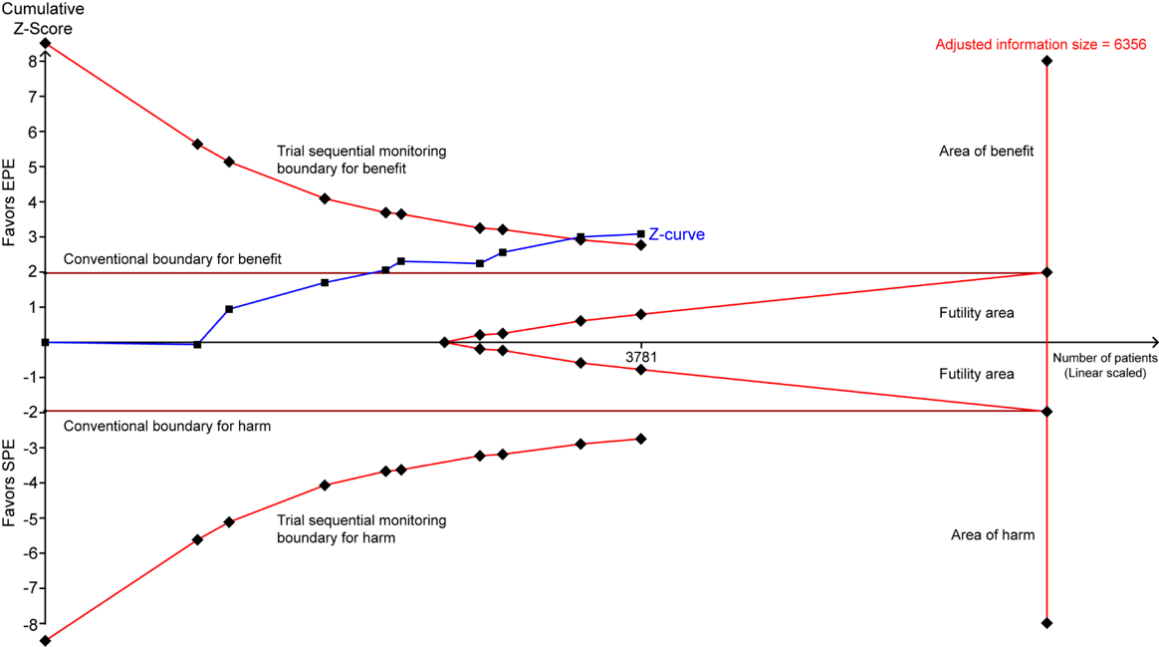
Trial sequential analysis of PDR. A diversity-adjusted information size of 6356 patients was calculated using alpha=.05 (2-sided), beta=.20 (power 80%), an anticipated relative risk reduction of 20%, and an event proportion of 30.23% in the SPE arm. The TSA-adjusted 95% CI for a relative risk of 1.31 is 1.10 to 1.56 (random effects model [DL]). TSA illustrated that the required information size was not achieved (adjusted information size=6356), however, that the cumulative z curve crossed the trial sequential monitoring boundary for benefit, showing that currently cumulative evidence is conclusive. PDR: polyp detection rate; EPE: enhanced patient education; SPE: standard patient education; TSA: trial sequential analysis; DL: DerSimonian and Laird.

**Figure 4:**
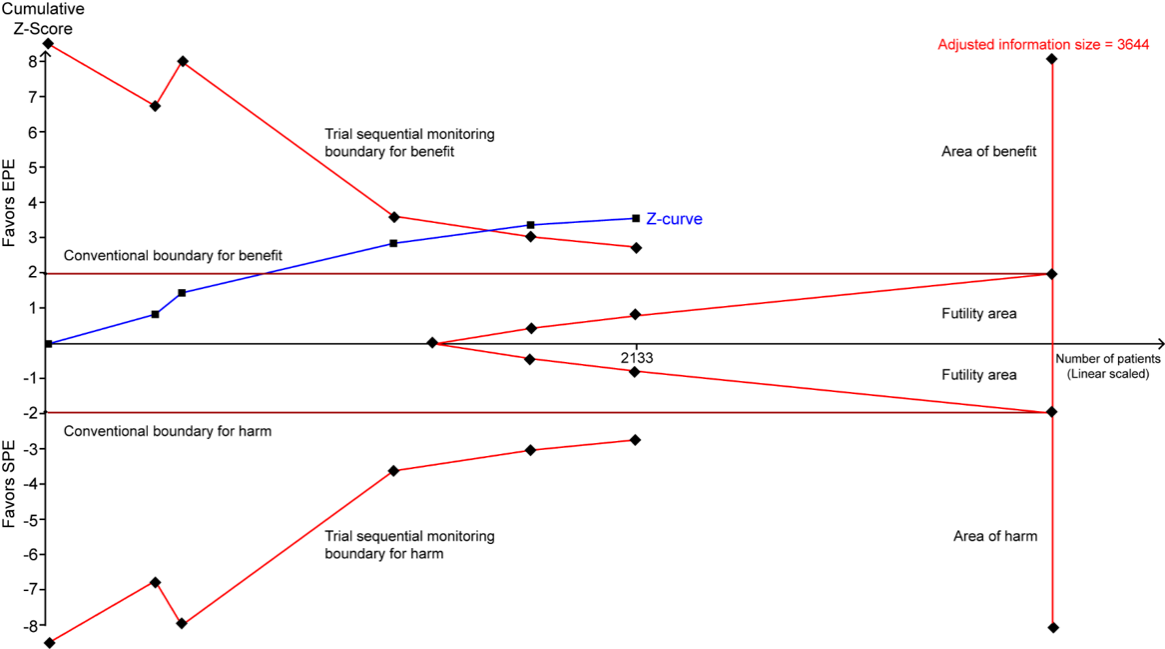
Trial sequential analysis of ADR. A diversity-adjusted information size of 3644 patients was calculated using alpha=.05 (2-sided), beta=.20 (power 80%), an anticipated relative risk reduction of 20%, and an event proportion of 16.49% in the SPE arm. The TSA-adjusted 95% CI for a relative risk of 1.37 is 1.15 to 1.64 (Random effects model [DL]). TSA illustrated that the required information size was not achieved (adjusted information size=3644), however, that the cumulative z curve crossed the trial sequential monitoring boundary for benefit, showing that currently cumulative evidence is conclusive. ADR: adenoma detection rate; EPE: enhanced patient education; SPE: standard patient education; TSA: trial sequential analysis; DL: DerSimonian and Laird.

TSA suggested that the accumulative Z-curve crossed the trial sequential monitoring boundary for benefit after the eighth trial of PDR (Figure 3) and after the fourth trial of ADR (Figure 4), showing that currently, the cumulative evidence for PDR and ADR is conclusive.

### Secondary Outcomes

**Figure 5:**
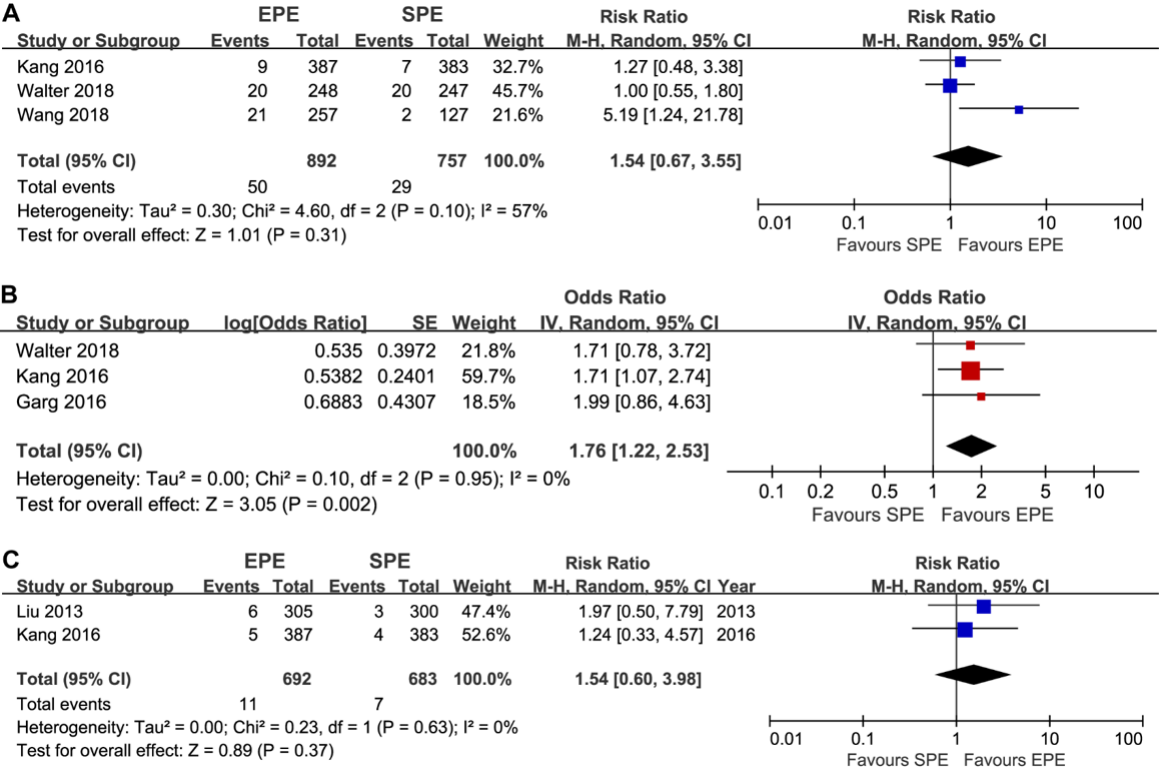
Meta-analysis of the effect of EPE on AADR (A), SSADR (B), and CDR (C). The summary effect estimates (odds ratio) for individual randomized controlled trials are indicated by blue or red rectangles (the size of the rectangle is proportional to the study weight), with the black horizontal lines representing 95% CIs. The overall summary effect estimate (OR) and 95% CI are indicated by the black diamond below. EPE: enhanced patient education; SPE: standard patient education; PDR: polyp detection rate; ADR: adenomas detection rate; RR: risk ratio; M-H: Mantel-Haenszel; IV: inverse variance.

Figure 5 delineated the summarized results of the secondary outcomes. Meta-analysis showed no significant difference in AADR (3 trials; 5.6% [50/892] of participants vs 3.8% [29/757] of participants; RR 1.54, 95% CI 0.67-3.55; *P*=.31; I^2^=57%) and CDR (2 trials; 1.59% vs 1.02%; RR 1.54, 95% CI 0.60-3.98; *P*=.37; I^2^=0%), and a significant difference in SSADR based on inverse-variance model (3 trials; OR 1.76, 95% CI 1.22-2.53; *P*<.05; I^2^=0%) between the EPE and standard patient education groups. Only 1 trial reported the ADR-plus, and adjusted estimate found a superior result in the EPE group (1 trial; RR 4.39, 95% CI 2.91-6.61; *P*<.001) [47]. One trial reported ADR according to segments of colon (right vs left) and did not find a significant difference between EPE and standard patient education interventions (right colon: RR 1.86, 95% CI 0.66-5.24 and left colon: RR 2.45, 95% CI 0.59-10.11) [41].

### Publication Bias

**Figure 6:**
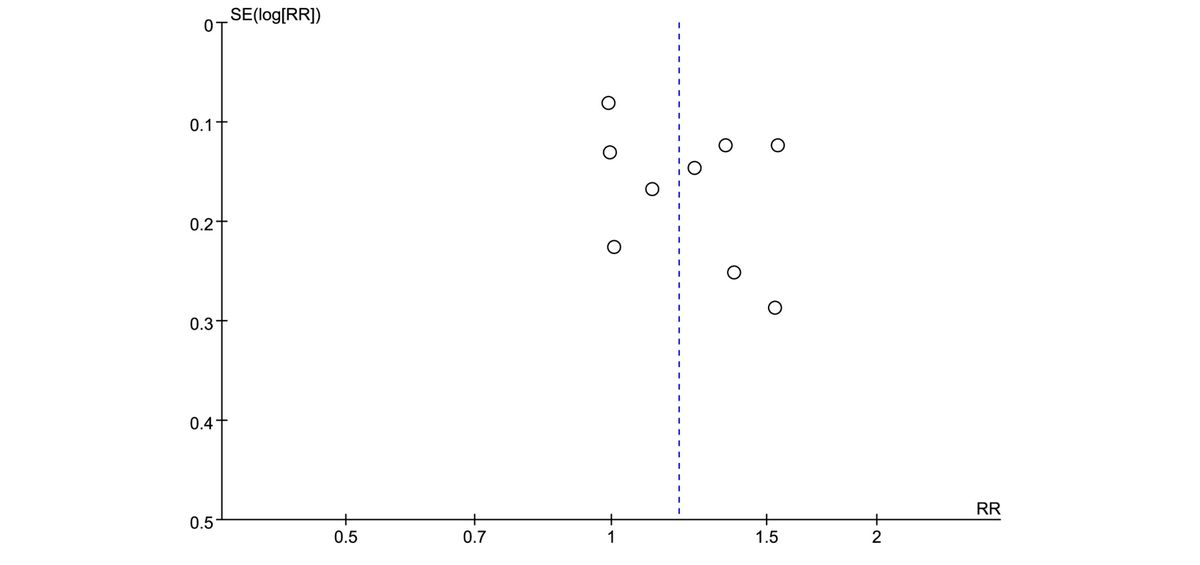
Funnel plot of PDR between the EPE and SPE groups. The vertical axis represents the standard error (SE) of effect size and x-axis indicates pooled risk ratio (RR). Symmetrical funnel plot indicates the absence of publication bias. PDR: polyp detection rate; EPE: enhanced patient education; SPE: standard patient education.

Although the accumulated number of analyzed trials for all outcomes was less than 10, we also constructed the funnel plot for PDR because 9 trials were incorporated into this outcome. The funnel plot is not symmetrical, and the publication bias cannot be excluded (Figure 6).

## Discussion

### Principal Findings

Adequate bowel preparation is a critical contributor to successful colonoscopy; however, the performance of traditional instructions of bowel preparation before colonoscopy in improving the quality of bowel preparation is not enough [10]. So, several enhanced patient instructions such as WeChat and SMS were developed to cover the shortcomings of the traditional instructions [2]. In this meta-analysis evaluating the detection rate of colonic polyps and adenomas, we found that EPE relatively increased PDR by 19% (>57/1000) and ADR by 37% (>61/1000), when compared with standard patient education before colonoscopy. Moreover, we also found that EPE was associated with increased SSADR (OR 1.76, 95% CI 1.22-2.53) and ADR-plus (RR 4.39, 95% CI 2.91-6.61). We found no evidence of statistical differences in AADR and CDR between the 2 groups. After performing a subgroup analysis, we found that EPE increased PDR by 21% in Asian patients and by 44% in mixed outpatients, and ADR by 39% and 37% in Western and Asian patients undergoing colonoscopy, respectively. Although our study found an increased PDR, ADR, SSADR, and ADR-plus in the EPE group, the pure efficacy of EPE alone in the detection of colonic polyps and adenomas needs to be further investigated because all patients in the EPE group also received standard patient education.

### Mechanism of Enhanced Patient Education

The EPE regimes were quite diverse in the 10 eligible trials, including visual aid, new visual aids, phone call, SMS, mobile apps, and multimedia education. We compared the essential characteristics of these EPE regimes with those of standard patient education (Multimedia Appendix 3). EPE has been shown to be effective in improving bowel preparation quality [2,19]. In this study, we further found that EPE increases the detection rate of colonic polyp and adenoma. EPE has several advantages including easy understanding of the education materials, easy access to the information of bowel preparation and dietary recommendations, more interactive approaches for seeking solutions to problems, or an additional approach to enhance correct memory compared with standard patient education [2]. After instructing patients with the EPE regime, the compliance of patients with instructions, including dietary recommendations and digestion of bowel preparation solutions, improves, which may be the possible reason for an increase in the colonic polyp and adenomas detection rate [2,9,42,43].

### Comparison With Other Studies

To date, 5 systematic reviews and meta-analyses have been performed to comprehensively investigate the impact of EPE on the quality of bowel preparation compared with standard patient education [2,16-19]. However, only 2 of those 5 evaluated the PDR as a secondary outcome [2,17]. Chang et al [37] conducted a meta-analysis of 3 trials to evaluate the impact of EPE on PDR in patients undergoing colonoscopy and found no significant difference between the 2 groups (RR 1.14, 95% CI 0.87-1.51; I^2^=79.1%). However, there are some limitations that need to be considered in this meta-analysis. First, only 3 trials including 1779 patients were analyzed, which may cause summary estimates to be inflated and thereby limit the strength of the inference that can be drawn due to inadequate accumulated sample size (adjusted information size=6356) [48]. Therefore, the finding of this study may not be considered as definitive. Furthermore, this meta-analysis did not consider other important colonoscopy quality metrics such as ADR and adenomas per positive participant [49], which greatly reduced the relevance for clinical decision.

Guo et al [2] performed an updated systematic review and meta-analysis after including recent trials to evaluate the efficacy of EPE for bowel preparation before colonoscopy. In this study, PDR was also evaluated as a secondary outcome, and a pooled estimate based on 5 trials did not detect a significant difference in PDR between the 2 groups (OR 1.25, 95% CI 0.93-1.68; *P*=.14). It must be noted, however, that there are also some flaws that need to be acknowledged in this study. First, although this study included more trials for final analysis [39,42,43,50,51], 2 studies that did not report appropriate data were inappropriately considered to be eligible [50,51]. As a result, the findings of this study must be interpreted cautiously. Similarly, this study did not also consider other important colonoscopy quality metrics [49].

Differences between our study and the 2 previous meta-analyses should be emphasized. In the 2 previous meta-analyses, limited number of eligible trials were accumulated for PDR, and since then, additional eligible trials with a high quality and large sample size have been published [9,40,41,44,46,47]. ADR is widely accepted as one of the objective colonoscopy quality metrics [52], and published evidence suggested that increased ADR was associated with decreased risk of post colonoscopy CRCs [53]. Moreover, ADR-plus was also suggested as one of the colonoscopy quality metrics with significant clinical relevance [28]. It is noted, however, that the previous meta-analyses only evaluated PDR, and other important quality metrics were not considered because of limited data, which reduced the clinical relevance of summary estimates. Our meta-analysis of 10 RCTs involving 4560 patients suggests that EPE is associated with an increase in PDR, ADR, ADR-plus, and SSADR. There were no significant differences in ADR and CDR. To test the robustness of summary results of primary outcomes, we performed a trial sequential meta-analysis to adjust the significance level and calculate the adjusted information size. Trial sequential analyses of both outcomes indicated that currently, cumulative evidences are conclusive. Moreover, subgroup meta-analyses found that EPE is only associated with increased PDR in Asian patients and ADR in all patients.

### Strengths and Limitations of This Study

Strengths of this review include a comprehensive literature search and the inclusion of multiple types of precursor polyps as outcomes. Moreover, we also performed a trial sequential meta-analysis for PDR and ADR. However, limitations of this meta-analysis should be acknowledged. First, 2 trials with a three-arm design were included, and we simply combined the data reported in the two positive groups according to the methodology recommended by the Cochrane Collaboration [25]. However, it may be rational to use the network meta-analysis to compare all interventions if more eligible trials can be captured. Second, most trials analyzed in our meta-analysis assigned patients to follow different diet restrictions such as clear liquid and low-residue diets, but we did not perform subgroup analysis according to this condition because 2 trials did not introduce the details of diet restriction [39,45]. It is worth mentioning, however, that previous meta-analyses have demonstrated comparable efficacy between clear liquid and low-residue diets for bowel preparation before colonoscopy [54,55]. Third, our previous meta-analysis detected no significant difference between the low- and traditional-volume polyethylene glycol regimens in bowel preparation [56]. Thus, bowel preparation regimen was not considered to be a factor for performing a subgroup analysis. However, prokinetic agents were used in some trials [39,40,42,43,46]. So, additional analyses should be performed when sufficient data on bowel preparation regimen can be obtained. Fourth, the start time of initiating education was different among all included trials. This factor and potential influence should be considered with caution. If possible, a further study should be designed to investigate the impact of starting time of education on the detection of colonic polyps or adenomas. Fifth, the EPE methods were quite diverse in the eligible RCTs; however, additional analysis cannot be performed to investigate which EPE method or which combination is the best for bowel preparation instruction because of insufficient data.

### Conclusions

In summary, current evidence indicates that there was a significant difference between EPE and standard patient education in PDR (RR 1.19, 95% CI 1.05-1.35; *P*<.05), ADR (RR 1.37, 95% CI 1.15-1.64; *P*<.001), ADR-plus (RR 4.39, 95% CI 2.91-6.61; *P*<.001), and SSADR (OR 1.76, 95% CI 1.22-2.53; *P*<.05). However, results for AADR (RR 1.54, 95% CI 0.67-3.55; *P*=.31), ADR-plus, and CDR (RR 1.54, 95% CI 0.60-3.98; *P*=.37) should be interpreted cautiously as data are still limited. Large-scale trials addressing this question may provide data better applicable to clinical practice.

## Appendix

Multimedia Appendix 1 Full search strategy.

Multimedia Appendix 2 Detailed summary of subgroup analyses.

Multimedia Appendix 3 Detailed summary of EPE regimes.

## Abbreviations

AADR: advanced adenoma detection rate
ADR: adenoma detection rate
CDR: cancer detection rate
CRC: colorectal cancer
OR: odds ratio
PDR: polyp detection rate
RCT: randomized controlled trial
RR: risk ratio
SSADR: sessile serrated adenoma detection rate
TSA: trial sequential analysis
