# Supplementary material for: Enhanced Patient Education for Colonic Polyp and Adenoma Detection: Meta-Analysis of Randomized Controlled Trials
Source: JMIR Mhealth Uhealth. 2020 Jun 1;8(6):e17372. doi: 10.2196/17372 (PMC7296415; doi:10.2196/17372)
Supplement: Multimedia Appendix 1 [file mhealth_v8i6e17372_app1.pdf]

Search strategy in PubMed

Search	Query
#11	Search (((((((education) OR educate) OR instruction) OR instruct)) AND (("Colonoscopy"[Mesh]) OR ((colonoscopy) OR colonoscopic))) AND (("Cathartics"[Mesh]) OR ((((bowel preparation) OR bowel cleansing) OR bowel evacuant) OR purgative))) AND ((((polyp) OR adenoma)) OR (("Polyps"[Mesh] OR "Adenomatous Polyps"[Mesh] OR "Colonic Polyps"[Mesh] OR "Intestinal Polyps"[Mesh]) OR "Adenoma"[Mesh])) Sort by: PublicationDate
#10	Search (((polyp) OR adenoma)) OR (("Polyps"[Mesh] OR "Adenomatous Polyps"[Mesh] OR "Colonic Polyps"[Mesh] OR "Intestinal Polyps"[Mesh]) OR "Adenoma"[Mesh]) Sort by: PublicationDate
#9	Search ("Polyps"[Mesh] OR "Adenomatous Polyps"[Mesh] OR "Colonic Polyps"[Mesh] OR "Intestinal Polyps"[Mesh]) OR "Adenoma"[Mesh] Sort by: PublicationDate
#8	Search (polyp) OR adenoma Sort by: PublicationDate
#7	Search ("Cathartics"[Mesh]) OR ((((bowel preparation) OR bowel cleansing) OR bowel evacuant) OR purgative) Sort by: PublicationDate
#6	Search (((bowel preparation) OR bowel cleansing) OR bowel evacuant) OR purgative Sort by: PublicationDate
#5	Search "Cathartics"[Mesh] Sort by: PublicationDate
#4	Search ("Colonoscopy"[Mesh]) OR ((colonoscopy) OR colonoscopic) Sort by: PublicationDate
#3	Search (colonoscopy) OR colonoscopic Sort by: PublicationDate
#2	Search "Colonoscopy"[Mesh] Sort by: PublicationDate
#1	Search (((education) OR educate) OR instruction) OR instruct Sort by: PublicationDate

Search strategy in Cochrane Library

#1 (education) OR (educate) OR (instruction) OR (instruct)
#2 (colonoscopy) OR (colonoscopic)
#3 MeSH descriptor: [Colonoscopy] explode all trees
#4 #2 OR #3
#5 (bowel preparation) OR (bowel cleansing) OR (bowel evacuant) OR (purgative)
#6 MeSH descriptor: [Cathartics] explode all trees
#7 #5 OR #6
#8 (polyp) OR (adenoma)
#9 MeSH descriptor: [Polyps] explode all trees
#10 MeSH descriptor: [Adenomatous Polyps] explode all trees
#11 MeSH descriptor: [Colonic Polyps] explode all trees
#12 MeSH descriptor: [Intestinal Polyps] explode all trees
#13 MeSH descriptor: [Adenoma] explode all trees
#14 #8 OR #9 OR #10 OR #11 OR #12 OR #13
#15 #1 AND #4 AND #7 AND #14

Search strategy in Embase

....

No. Query Results

#17. #1 AND #4 AND #8 AND #16

#16. #9 OR #10 OR #11 OR #12 OR #13 OR #14 OR #15

#15. 'rectum polyp'/exp

#14. 'adenoma'/exp

#13. 'colon polyp'/exp

#12. 'colon polyp'/exp

#11. 'adenomatous polyp'/exp

#10. 'polyp'/exp

#9. 'polyp'/exp OR polyp OR 'adenoma'/exp OR adenoma

#8. #5 OR #6 OR #7

#7. 'intestine preparation'/exp

#6. 'laxative'/exp

#5. 'bowel preparation'/exp OR 'bowel preparation' OR (('bowel'/exp OR bowel) AND ('preparation'/exp OR preparation)) OR 'bowel cleansing'/exp OR 'bowel cleansing' OR (('bowel'/exp OR bowel) AND ('cleansing'/exp OR cleansing)) OR 'bowel evacuant'/exp OR 'bowel evacuant' OR (('bowel'/exp OR bowel) AND evacuant) OR 'purgative'/exp OR purgative

#4. #2 OR #3

#3. 'colonoscopy'/exp

#2. 'colonoscopy'/exp OR colonoscopy OR colonoscopic

#1. 'education'/exp OR education OR educate OR instruction OR instruct

....
