# Supplementary material for: Enhanced Patient Education for Colonic Polyp and Adenoma Detection: Meta-Analysis of Randomized Controlled Trials
Source: JMIR Mhealth Uhealth. 2020 Jun 1;8(6):e17372. doi: 10.2196/17372 (PMC7296415; doi:10.2196/17372)
Supplement: Multimedia Appendix 2 [file mhealth_v8i6e17372_app2.pdf]

Study or Subgroup	EPE Events	EPE Total	SPE Events	SPE Total	Weight	Risk Ratio M-H, Random, 95% CI	Year
1. Western							
Calderwood 2011	182	477	189	492	19.9%	0.99 [0.85, 1.17]	2011
Garg 2016	23	48	16	46	5.5%	1.38 [0.84, 2.26]	2016
Walter 2018	100	248	74	247	14.2%	1.35 [1.06, 1.72]	2018
**Subtotal (95% CI)**		**773**		**785**	**39.6%**	**1.17 [0.92, 1.50]**	
Total events	305		279				
Heterogeneity: Tau² = 0.03; Chi² = 5.00, df = 2 (P = 0.08); I² = 60%							
Test for overall effect: Z = 1.30 (P = 0.20)							
2. Asian							
Tae 2012	55	102	53	98	13.5%	1.00 [0.77, 1.29]	2012
Liu 2013	116	305	74	300	14.1%	1.54 [1.21, 1.97]	2013
Lee 2015	101	253	44	137	11.9%	1.24 [0.93, 1.66]	2015
Park 2016	34	250	34	252	6.5%	1.01 [0.65, 1.57]	2016
Cho 2017	23	71	15	71	4.4%	1.53 [0.87, 2.69]	2017
Wang 2018	81	257	36	127	9.9%	1.11 [0.80, 1.55]	2018
**Subtotal (95% CI)**		**1238**		**985**	**60.4%**	**1.21 [1.03, 1.43]**	
Total events	410		256				
Heterogeneity: Tau² = 0.01; Chi² = 7.66, df = 5 (P = 0.18); I² = 35%							
Test for overall effect: Z = 2.31 (P = 0.02)							
**Total (95% CI)**		**2011**		**1770**	**100.0%**	**1.19 [1.05, 1.35]**	
Total events	715		535				
Heterogeneity: Tau² = 0.01; Chi² = 13.77, df = 8 (P = 0.09); I² = 42%							
Test for overall effect: Z = 2.67 (P = 0.008)							
Test for subgroup differences: Chi² = 0.05, df = 1 (P = 0.82), I² = 0%							

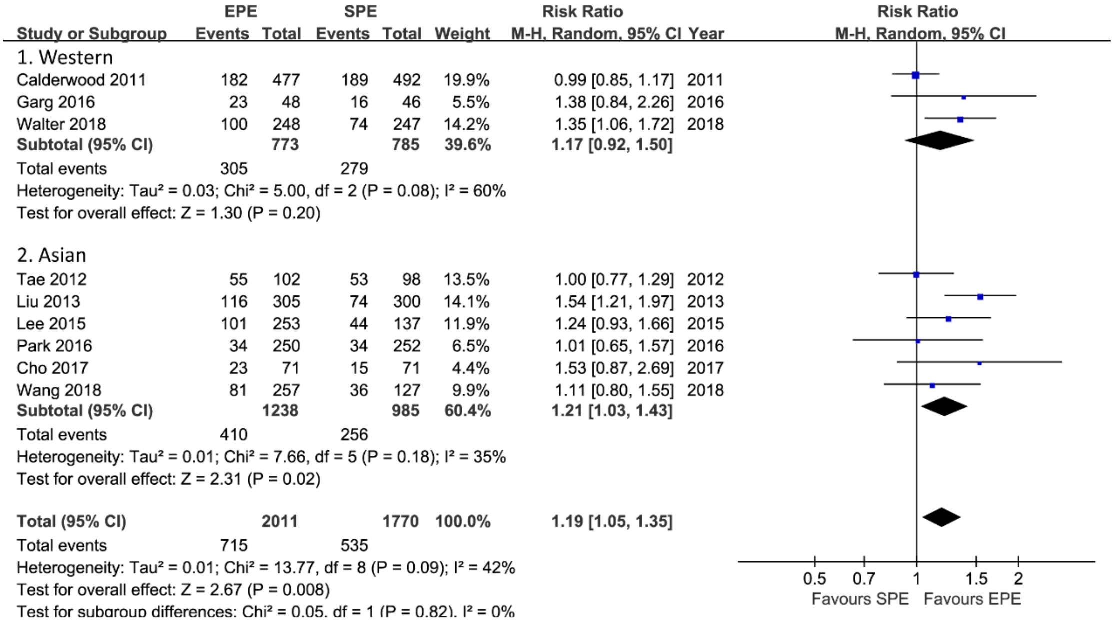

Figure S1. Subgroup analysis of PDR between the EPE and SPE groups based on geographical regions: (1) Western and (2) Asian. This pooled result indicated a statistical difference regarding PDR for Asian patients between EPE and SPE groups. The summary effect estimates (risk ratio, RR) for individual randomized controlled trial (RCT) are indicated by blue rectangles (the size of the rectangle is proportional to the study weight), with the black horizontal lines representing 95% confidence intervals (CIs). The overall summary effect estimate (RR) and 95% confidence interval are indicated by the black diamond below. PDR, polyp detection rate; EPE, enhanced patient education; SPE, standard patient education.

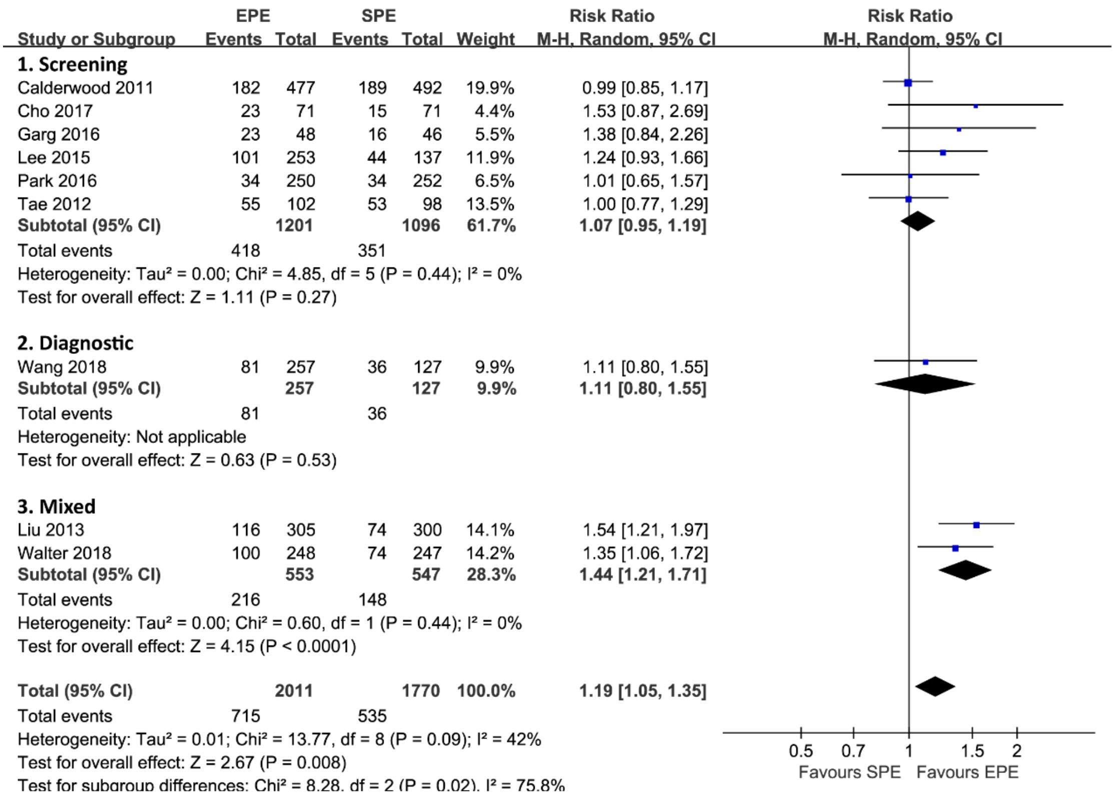

Figure S2. Subgroup analysis of PDR between EPE and SPE groups based on indications: (1) Screening colonoscopy, (2) dignostic colonoscopy and (3) Mixed including screening, surveillance and diagnostic colonoscopy. This pooled result indicated a statistical difference regarding PDR for patients undergoing mixed colonoscopies between EPE and SPE groups. The summary effect estimates (risk ratio, RR) for individual randomized controlled trial (RCT) are indicated by blue rectangles (the size of the rectangle is proportional to the study weight), with the black horizontal lines representing 95% confidence intervals (CIs). The overall summary effect estimate (RR) and 95% confidence interval are indicated by the black diamond below. PDR, polyp detection rate; EPE, enhanced patient education; SPE, standard patient education.

Study or Subgroup	EPE Events	EPE Total	SPE Events	SPE Total	Weight	Risk Ratio M-H, Random, 95% CI
**1. Split-dose**						
Lee 2015	101	253	44	137	14.9%	1.24 [0.93, 1.66]
Park 2016	34	250	34	252	7.0%	1.01 [0.65, 1.57]
Tae 2012	55	102	53	98	17.8%	1.00 [0.77, 1.29]
Walter 2018	100	248	74	247	19.2%	1.35 [1.06, 1.72]
Wang 2018	81	257	36	127	11.7%	1.11 [0.80, 1.55]
**Subtotal (95% CI)**		**1110**		**861**	**70.7%**	**1.16 [1.02, 1.32]**
Total events	371		241			
Heterogeneity: Tau² = 0.00; Chi² = 3.47, df = 4 (P = 0.48); I² = 0%						
Test for overall effect: Z = 2.21 (P = 0.03)						
**2. Single-dose**						
Cho 2017	23	71	15	71	4.5%	1.53 [0.87, 2.69]
Garg 2016	23	48	16	46	5.7%	1.38 [0.84, 2.26]
Liu 2013	116	305	74	300	19.1%	1.54 [1.21, 1.97]
**Subtotal (95% CI)**		**424**		**417**	**29.3%**	**1.51 [1.23, 1.85]**
Total events	162		105			
Heterogeneity: Tau² = 0.00; Chi² = 0.16, df = 2 (P = 0.92); I² = 0%						
Test for overall effect: Z = 3.97 (P < 0.0001)						
**Total (95% CI)**		**1534**		**1278**	**100.0%**	**1.25 [1.11, 1.41]**
Total events	533		346			
Heterogeneity: Tau² = 0.00; Chi² = 8.29, df = 7 (P = 0.31); I² = 16%						
Test for overall effect: Z = 3.56 (P = 0.0004)						
Test for subgroup differences: Chi² = 4.63, df = 1 (P = 0.03), I² = 78.4%						

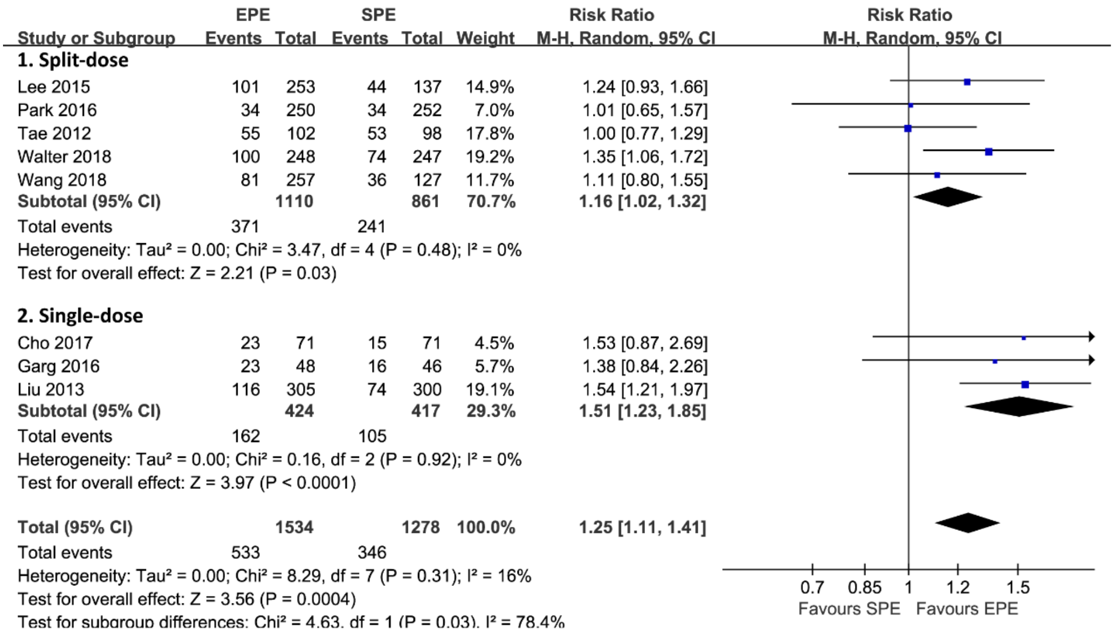

Figure S3. Subgroup analysis of PDR between EPE and SPE groups based on administration of colon cleans solutions: (1) split-dose and (2) single-dose. This pooled result indicated a statistical difference regarding PDR for all patients ingesting solutions with split-dose and single-dose between EPE and SPE groups. The summary effect estimates (risk ratio, RR) for individual randomized controlled trial (RCT) are indicated by blue rectangles (the size of the rectangle is proportional to the study weight), with the black horizontal lines representing 95% confidence intervals (CIs). The overall summary effect estimate (RR) and 95% confidence interval are indicated by the black diamond below. PDR, polyp detection rate; EPE, enhanced patient education; SPE, standard patient education.

Study or Subgroup	EPE Events	EPE Total	SPE Events	SPE Total	Weight	Risk Ratio M-H, Random, 95% CI	Year
**1. Western**							
Garg 2016	16	48	9	46	6.3%	1.70 [0.84, 3.46]	2016
Walter 2018	71	248	53	247	33.0%	1.33 [0.98, 1.82]	2018
**Subtotal (95% CI)**		**296**		**293**	**39.3%**	**1.39 [1.04, 1.84]**	
Total events	87		62				
Heterogeneity: Tau² = 0.00; Chi² = 0.38, df = 1 (P = 0.54); I² = 0%							
Test for overall effect: Z = 2.26 (P = 0.02)							
**2. Asian**							
Lee 2015	61	253	28	137	20.1%	1.18 [0.79, 1.75]	2015
Kang 2016	72	387	46	383	27.0%	1.55 [1.10, 2.18]	2016
Wang 2018	51	257	19	127	13.6%	1.33 [0.82, 2.15]	2018
**Subtotal (95% CI)**		**897**		**647**	**60.7%**	**1.37 [1.09, 1.72]**	
Total events	184		93				
Heterogeneity: Tau² = 0.00; Chi² = 1.06, df = 2 (P = 0.59); I² = 0%							
Test for overall effect: Z = 2.69 (P = 0.007)							
**Total (95% CI)**		**1193**		**940**	**100.0%**	**1.37 [1.15, 1.64]**	
Total events	271		155				
Heterogeneity: Tau² = 0.00; Chi² = 1.45, df = 4 (P = 0.84); I² = 0%							
Test for overall effect: Z = 3.51 (P = 0.0004)							
Test for subgroup differences: Chi² = 0.01, df = 1 (P = 0.94), I² = 0%							

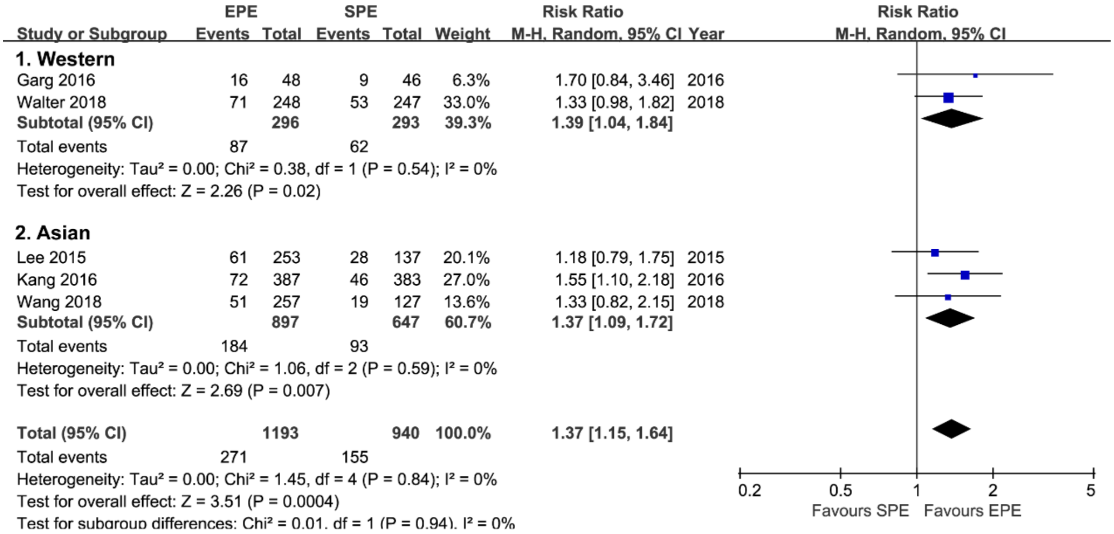

Figure S4. Subgroup analysis of ADR between the EPE and SPE groups based on geographical regions: (1) Western and (2) Asian. This pooled result indicated a statistical difference regarding PDR for Western and Asian patients between EPE and SPE groups. The summary effect estimates (risk ratio, RR) for individual randomized controlled trial (RCT) are indicated by blue rectangles (the size of the rectangle is proportional to the study weight), with the black horizontal lines representing 95% confidence intervals (CIs). The overall summary effect estimate (RR) and 95% confidence interval are indicated by the black diamond below. ADR, adenoma detection rate; EPE, enhanced patient education; SPE, standard patient education.

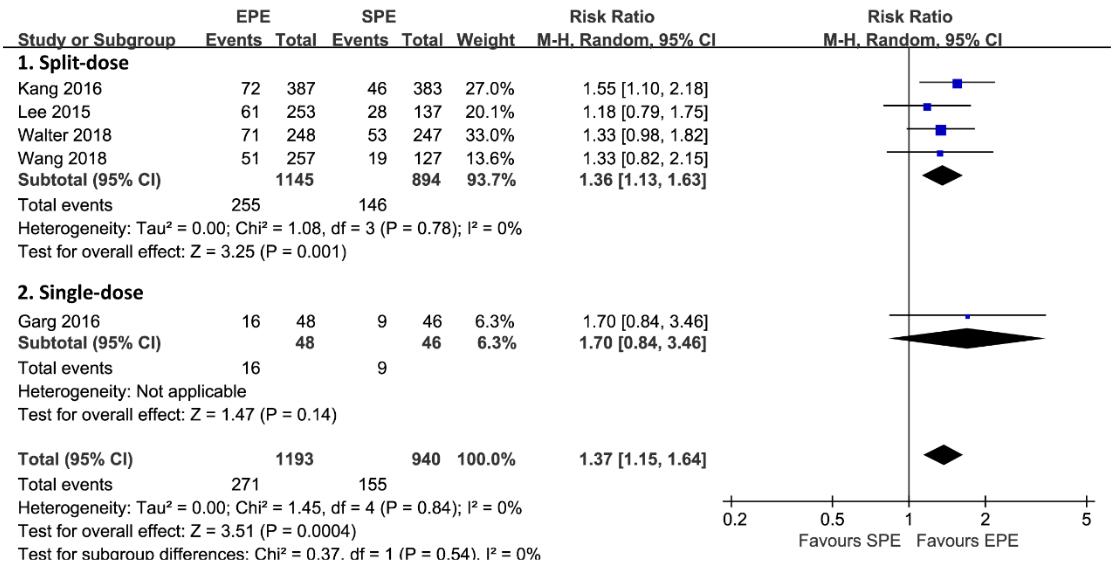

Figure S5. Subgroup analysis of ADR between EPE and SPE groups based on administration of colon cleans solutions: (1) split-dose and (2) single-dose. This pooled result indicated a statistical difference regarding ADR for all patients ingesting solutions with split-dose and single-dose between EPE and SPE groups. The summary effect estimates (risk ratio, RR) for individual randomized controlled trial (RCT) are indicated by blue rectangles (the size of the rectangle is proportional to the study weight), with the black horizontal lines representing 95% confidence intervals (CIs). The overall summary effect estimate (RR) and 95% confidence interval are indicated by the black diamond below. ADR, adenoma detection rate; EPE, enhanced patient education; SPE, standard patient education.
