# Supplementary material for: Enhanced Patient Education for Colonic Polyp and Adenoma Detection: Meta-Analysis of Randomized Controlled Trials
Source: JMIR Mhealth Uhealth. 2020 Jun 1;8(6):e17372. doi: 10.2196/17372 (PMC7296415; doi:10.2196/17372)
Supplement: Multimedia Appendix 3 [file mhealth_v8i6e17372_app3.docx]

**Multimedia Appendix 3. Comparison of the characteristics of EPE regimes of all eligibel trials.**

| **EPE regime** | **Description** | **Possible advantages compared with SPE** | | | |
| --- | --- | --- | --- | --- | --- |
|  |  | **Easy understanding** | **Easy accessing** | **More interactive** | **Augmenting memory** |
| ***Visual aid****^[39]^* | Providing patients with several endoscopic images demonstrating poor and good bowel preparation and some colonic polyps and adenomas. | Yes |  |  |  |
| ***New visual aids****^[45]^* | Providing cartoons with several pictures delineating poor and good bowel preparation, dangerous complications during colonoscopy and foods to avoid. | Yes |  |  |  |
| ***Phone call****^[42,43]^* | Calling patients to emphasize the importance of bowel preparation, the directions for use and side effects of purgatives, the proper food type, and the start time and encouraging patients to contact the investigator if they had any questions. |  |  | Yes | Yes |
| ***Mobile app****^[9,40,47]^* | Using a mobile app such as WeChat to introduce all information related to the colonoscopy appointment time, improtance of bowel preparation quality, method of ingesting bowel-cleansing agents, dietary recommendations, possible adverse events during bowel preparation and colonoscopy etc. | Yes | Yes |  |  |
| ***SMS****^[42,46,47]^* | Sending repeated text-message to patients to remind the schedule colonoscopy appointment time, dietary restrictions, adequate timing of purgative intake and answering the questions. |  |  | Yes | Yes |
| ***Multimedia education****^[41,44]^* | Using multiple education approaches to provide bowel preparation related information, including visual aid describing bowel preparation quality and types of polyps and cancers in the colon, audio-visual education introducing the importance of screening or surveillance colonoscopy, importance of adequate bowel preparation, and brochure addressing the importance of bowel preparation. | Yes | Yes |  | Yes |

EPE: enhanced patient education; SPE: standard patient education; SMS: short message service.
